# European headache federation consensus on technical investigation for primary headache disorders

**DOI:** 10.1186/s10194-016-0596-y

**Published:** 2016-02-09

**Authors:** D. D. Mitsikostas, M. Ashina, A. Craven, H. C. Diener, P. J. Goadsby, M. D. Ferrari, C. Lampl, K. Paemeleire, J. Pascual, A. Siva, J. Olesen, V. Osipova, P. Martelletti, on behalf of EHF committee

**Affiliations:** Neurology Department, Athens Naval Hospital, Athens, Greece; Danish Headache Center, Rigshospitalet Glostrup, Faculty of Health and Medical Sciences, University of Copenhagen, Copenhagen, Denmark; European Headache Alliance, President, Dublin, Ireland; Department of Neurology, University Hospital Essen, University Duisburg-Essen, Essen, Germany; Basic and Clinical Neurosciences, Institute of Psychiatry, Psychology and Neuroscience, and King’s Clinical Research Facility, Kings College London, Wellcome Foundation Building, King’s College Hospital, London, SE5 9PJ UK; Center for Proteomics and Metabolomics, Leiden University Medical Center, Albinusdreef 2, 2333 ZA Leiden, The Netherlands; Medical Headache Center, Hospital Sisters of Mercy, Seilerstaette Linz, Linz, 4020 Austria; Department of Neurology, Ghent University Hospital, Ghent, Belgium; University Hospital Marqués de Valdecilla and IDIVAL, 39011 Santander, Spain; Department of Neurology, Cerrahpasa School of Medicine, Istanbul University, Millet Cad, 34390 Capa/Istanbul, Turkey; Danish Headache Centre and Department of Neurology, Rigshospitalet, Glostrup, Faculty of Health and Medical Sciences, University of Copenhagen, Copenhagen, Denmark; Department of Neurology, First Moscow State Medical University, Moscow, Russia; Department of Clinical and Molecular Medicine, Sapienza University, Rome, Italy

**Keywords:** Primary headache disorders, Migraine, Tension-type headache, TACs, Consensus, Diagnostic tests, Brain MRI

## Abstract

The diagnosis of primary headache disorders is clinical and based on the diagnostic criteria of the International Headache Society (ICHD-3-beta). However several brain conditions may mimic primary headache disorders and laboratory investigation may be needed. This necessity occurs when the treating physician doubts for the primary origin of headache. Features that represent a warning for a possible underlying disorder causing the headache are new onset headache, change in previously stable headache pattern, headache that abruptly reaches the peak level, headache that changes with posture, headache awakening the patient, or precipitated by physical activity or Valsalva manoeuvre, first onset of headache ≥50 years of age, neurological symptoms or signs, trauma, fever, seizures, history of malignancy, history of HIV or active infections, and prior history of stroke or intracranial bleeding. All national headache societies and the European Headache Alliance invited to review and comment the consensus before the final draft. The consensus recommends brain MRI for the case of migraine with aura that persists on one side or in brainstem aura. Persistent aura without infarction and migrainous infarction require brain MRI, MRA and MRV. Brain MRI with detailed study of the pituitary area and cavernous sinus, is recommended for all TACs. For primary cough headache, exercise headache, headache associated with sexual activity, thunderclap headache and hypnic headache apart from brain MRI additional tests may be required. Because there is little and no good evidence the committee constructed a consensus based on the opinion of experts, and should be treated as imperfect.

## Main text

Headache is a symptom in the main rather a condition. Only when headache attacks fulfill specific diagnostic criteria consistently does a primary headache disorder occur [1]. Accompanying symptoms are important together with the particular headache characteristics including pain severity, duration, quality and location. In most cases headache is primary but secondary headache disorders may be related to life threatening conditions. They may respond to common analgesics and mimic primary ones a lot. Diagnostic tests are necessary therefore when the treating physician doubts for the primary origin of headache. Up-to-date there is no official recommendation for these tests, although headache remains the commonest presenting symptom in people asking medical consultation. To bridge this gap European Headache Federation (EHF) appointed an internal and external committee to prepare a consensus on the diagnostic testing that primary headache disorders may require. The procedure followed was consisted of three phases. In phase one members of the Executive Board of EHF (internal committee: DDM, AS, CL, KP, VO and PM) prepared the first draft that was send to all National European Headache Societies for review. Thirteen National Headache Societies replied (40 %) with comments. European Headache Alliance also participated in this review phase. Based on their comments and suggestions draft 2 was edited (phase 2) that was applied for review in a group of distinguished headache specialists that EHF appointed as the external subcommittee (MA, HCD, MDF, PJG, JP, and JO). After fulfilling all comments into one manuscript the final draft of the consensus was arranged (phase 3), which is presented in the Appendix. Table 1 summarizes the principles.

**Table 1:** Tests recommended for primary headache disorders

ICHD-IIIb code	Disorder	Tests
1	Migraine	
1.1	Migraine without aura	None
	Frequent episodic migraine	Brain MRI^a^
		Carodit ultrasound or MRA^a^
		ESR^a^
1.2	Migraine with aura	Brain MRI^a^
1.2.2	Migraine with brainstem aura	Brain MRI & MRA
		EEG^a^
		Carotid and vertebral arteries ultrasound/or CT or MRA^a^
		Genetic evaluation^a^
1.3	Chronic migraine	Brain MRI Gd & MRV^a^
		Fundoscopy^a^
		Lumbar puncture^a^
		Polysomnography^a^
1.4	Complications of migraine	Brain MRI
1.4.1/2	Persistence of aura symptoms	Emergency brain CT or MRI
		Carotid and vertebral arteries ultrasound/or CT or MRA
		ESR^a^
1.4.3	Migrainous infarction	Emergency brain CT or MRI
		Carotid and vertebral arteries ultrasound/or CT or MRA^a^
		ESR^a^
1.4.3	Migraine aura-triggered seizures	Repetitive EEGs or video EEG
1.5	Probable migraine	Brain MRI^a^
1.6	Episodic syndromes that may be associated with migraine	Gastric work-up
2	TTH	
2.1	Infrequent TTH	None
2.2 and 3	Frequent TTH and Chronic TTH	Brain MRI MRI Gd & MRV^a^
		Fundoscopy^a^
		Lumbar puncture^a^
		Polysomnography^a^
3	TACs (all)	Brain MRI
		Brain MRA and Carotid and vertebral arteries ultrasound/or CT or MRA^a^
		Pituitary function testing^a^
3.1	Cluster headache	+Polysomnography^a^
3.2	Paraxysmal Hemicrania	Brain MRI and MRA and Carotid and vertebral arteries ultrasound/or CT or MRA^a^
3.3	Suna & Sunct	+ High resolution MRI of brainstem
3.4	Hemicrania Continua	Brain MRI
		Brain MRA and Carotid and vertebral arteries ultrasound/or CT or MRA^a^
4	Other primary headache disorders	
4.1	Primary cough headache	Brain MRI
		Cranio-cervical and brain MRA^a^
4.2	Primary exercise headache	Brain MRI and MRA/MRV
		Lumbar puncture^a^
		Carotid & vertebral ultrasound, or CT or MRA
		Cardiological evaluation ^a^
4.3	Primary headache associated with sexual activity	Brain MRI, MRA/MRV
		Carotid and vertebral arteries ultrasound/or CT or MRA
		Lumbar puncture^a^
		ESR, CRP^a^
4.4	Primary thunderclap headache	Brain MRI, MRA/MRV
		Carotid and vertebral arteries ultrasound/or CT or MRA
		Lumbar puncture^a^
		ESR, CRP^a^
4.5	Cold-stimulus headache	None
4.6	External-pressure headache	None
4.7	Primary stabbing headache	Brain MRI, MRA/MRV
		Carotid and vertebral arteries ultrasound/or CT or MRA
		Lumbar puncture^a^
		ESR, CRP^a^
4.8	Nummular headache	Brain MRI, ESR, ANF and RF
4.9	Hypnic headache	Brain MRI, ESR, CRP
		Polysomnography
		24-hour blood pressure monitoring
4.10	New daily persistent headache	Brain MRI, MRA
		Lumbar puncture^a^

^a^Indicates specific conditions

## Appendix

### 1. Introduction

The following recommendations concern adults with headache as the leading symptom over the age of 18.

The majority of patients presenting with headache suffer from primary headache disorders. The diagnosis of primary headache disorders is mainly clinical and based on both history taking and a normal clinical neurological examination. However, medical practice changes with time, and laboratory investigation may be needed to exclude even very rare conditions in order to protect better patients and improve headache management. Investigations are required when the treating physician is in doubt whether they are dealing with a primary headache disorder. Clinical features that warn of a possible underlying disorder causing headache include:

1. Headache that peaks in severity in less than five minutes
2. New headache type versus a worsening of a previous headache
3. Change in previously stable headache pattern
4. Headache that changes with posture (e.g. standing up)
5. Headache awakening the patient
6. Headache precipitated by physical activity or Valsalva manoeuvre (e.g. coughing, laughing, straining)
7. First onset ≥50 years of age
8. Neurological symptoms or signs
9. Trauma
10. Fever
11. Seizures
12. History of malignancy
13. History of HIV or active infections

Physicians should also consider that identification of a comorbid disorder does not necessarily imply that the headache can be attributed to the condition. A secondary headache disorder can be diagnosed when one of the following features is present [1]:

1. Another disorder documented to be able to cause headache has been diagnosed or is suspected.
2. Headache has developed in close temporal relation to the onset of the presumed causative disorder.
3. Headache has significantly worsened in parallel with worsening of the presumed causative disorder and/or headache has significantly improved in parallel with improvement of the presumed causative disorder.
4. Headache has characteristics typical of the causative disorder and other evidence exists of causation.
5. Headache fulfills criteria for primary headache, but has a markedly late onset.

Additionally, for those patients who present with a history of headache attacks with unchanged features and have not been investigated previously, the following recommendations are appropriate. When the diagnosis of a primary headache has been made for years, but headache features change or additional general symptoms occur or neurological examination changes, apart from brain MRI additional investigation may be required according to the treating physicians’ decision and the patient’s specific clinical presentation. If a patient asks for laboratory evaluation outside the above or the following recommendations, or the treating physician feels that there is need for such an evaluation, the committee recommends dealing with such situations on a case by case basis and being aware of cost issues. Local reimbursement regulations should be considered as well. 

The following recommendations are based on the opinion of experts, and should be treated as imperfect. The recommendations are supported by a literature search done for each specific primary headache disorder covering articles published the last 10 years.

### 2. Recommendations

The following recommendations cover the first onset of a primary headache disorder. Recommendations follow the classification of the International Classification of Headache Disorders (ICHD-3-beta) [1].

### 1. Migraine

#### 1.1 Migraine without aura

Generally no investigation is required, in particular when no preventive treatment is needed. For episodic migraine without aura that needs preventive treatment, some experts recommend brain MRI when three first line preventive treatments fail [2, 3]. In the presence of a normal neurological examination this may not be necessary. Additionally, carotid ultrasound or MRA of the carotid and vertebral arteries with fat saturated T1 sequences are recommended by some experts when migraine occurs in patients over 50 years of age for the first time, or in the presence of cerebrovascular comorbidity [4]. Again in the absence of non-headache disorder neurological symptoms or signs, this is not a universal practice. In the appropriate clinical setting, a physician should consider giant cell arteritis, with symptoms such as pain upon mastication, visual disturbance, suspect temporal artery upon palpation, and perform recommended testing.

#### 1.2 Migraine with aura

Brain MRI is suggested for almost all migraine with aura sub-forms based solely on their rarity and concern with accurate diagnosis. For migraine typical aura, unless there are other reasons, investigation is not recommended however. In migraine with brainstem aura some experts would recommend brain MRA. If brain MRI or MRA shows abnormalities the treating physician will decide whether additional investigation is required to rule out potentially related vascular (AV malformations, carotid dissection) or in some cases, heart conditions, such as patent foramen ovale (PFO), may be considered although there are no compelling data to treat PFO for the indication of migraine. Unless epilepsy is being considered, EEG has no place in the evaluation of primary headache disorders. Occipital seizures are noted to produce visual disturbance that is phenotypically distinct from migraine [5, 6]. Prolonged video EEG is never useful to investigate migraine aura, unless the clinician is considering epilepsy as a diagnosis. Transient ischemic attacks should be ruled out, when the onset of symptoms is abrupt, symptoms are exclusively negative (e.g. hemianopsia) or the aura is prolonged or very short in duration [1]. Angio-CT or MRA of carotid and vertebral arteries with fat saturated T1 sequences is recommended by some experts when migraine aura occurs for the first time in patients over 50 years of age, or in the presence of cerebro-vascular comorbidity [4], although when aura typical a case can be made not to investigate. For hemiplegic migraine, mutation screening of CACNA1A (FHM1), ATP1A2 (FHM2) and SCN1A (FHM3) is suggested although it has neither phenotypic nor therapeutic application at this time [7].

#### 1.3 Chronic migraine

When there is the typical history of gradual increase of headache frequency over the years and ICHD-3-beta criteria for chronic migraine are satisfied, there is no need for additional investigations. Funduscopy should be a routine part of the evaluation of all newly presenting patients with a headache disorders and clinicians should be particularly mindful of its importance in patients who are over-weight. MR venography of the brain is recommended in the presence of symptoms suggestive of intracranial hypertension, such as pulsatile tinnitus and transient visual obscurations, or increasing pain with Valsalva maneuver or daily morning headache that spontaneously resolves during the day [8]. Lumbar puncture with CSF manometry, to look for idiopathic intracranial hypertension (IIH) without papilledema, is not recommended, unless clinical signs indicate IIH [9]. In all chronic migraine patients when intracranial hypertension is established, or suspected, metabolic, toxic and endocrine disorders need to be considered by appropriate tests. Polysomnography may be recommended in the presence of signs and symptoms (e.g. strictly morning headache) suggestive of obstructive sleep apnea (OAS), although there is data indicating that migraine is not related to OAS in the general population [10]. Detecting comorbid psychiatric disorders is appropriate in all patients.

#### 1.4 Complications of migraine

In the presence of persistence of aura symptoms the admission to the emergency room and urgent brain DWI MRI is recommended to exclude the presence of brain ischaemia. Generally, persistent aura without infarction and migrainous infarction require both brain MRI, MRA and MRV, plus further investigation for thrombophilia and other risk factors for brain thrombosis, including patent PFO, although the causative relation of PFO and migraine is questionable [11] (not all experts agree there is enough data to support testing for PFO). For migraine aura-triggered seizures, repetitive EEGs or long lasting video-EEG recording is recommended [5, 6].

#### 1.5 Probable Migraine with aura

For probable migraine with aura, the same investigations such as those in section1.2 Migraine with aura are recommended.

#### 1.6 Episodic syndromes that may be associated with migraine

For episodic syndromes that may be associated with migraine, extensive gastrointestinal work-up is recommended. For benign paroxysmal vertigo and torticollis brain MRI may be needed, according to treating physician’s opinion.

### 2. Tension-type headache

#### 2.1 Infrequent TTH

No investigation is needed.

#### 2.2 Frequent TTH and 2.3 Chronic TTH

MRI brain may be useful when three first line preventive treatments fail [12]. For overweight or obese individuals paraclinical investigation as in 1.3 (Chronic migraine).

#### 2.4 Probable TTH

Infrequent probable TTH as 2.1; frequent and chronic probable TTH, as 2.2.

### 3. Trigeminal autonomic cephalalgias (TACs)

Brain MRI with detailed study of the pituitary area and cavernous sinus, is recommended for all TACs. When three consecutive preventive treatments fail additional MRA brain and carotid/vertebral arteries may be required and in the presence of a (partial) Horner’s syndrome, additional imaging of the apex of the lung may be warranted, especially in smokers [13]. Pituitary function testing should be considered in refractory TAC patients in addition [14]. Comorbidity with trigeminal neuralgia (TAC-tic syndrome) should be ruled out in cases of refractory TACs.

#### 3.1 Cluster headache

The same exams as in section 3. In addition polysomnography may be needed when patients are snoring and pituitary function testing should be considered in refractory cluster headache (CH) patients [15, 16]. Restless legs syndrome may also be co-exist with cluster headache, but this does not seem to affect CH management [17]. During polysomnography restless legs syndrome could be documented and treated appropriately. Despite the above recommendations, CH patients with long history of CH bouts separated by asymptomatic periods that respond to preventive treatment may not need neuroimaging.

#### 3.2 Paroxysmal hemicrania

The same exams as in section 3.

#### 3.3 SUNCT (Short-lasting unilateral neuralgiform headache attacks with conjunctival injection and tearing) and SUNA

A SUNCT syndrome may be hard to differentiate from V1 trigeminal neuralgia. Evidence for neurovascular compression can be sought by high-resolution MRI sequences of the brain stem and cranial nerves [18], although if clinicians are unsure about the diagnosis it is preferable to involve expert colleagues before obtaining a neurosurgical opinion. SUNCT has also been described in multiple sclerosis [19, 20].

#### 3.4 Hemicrania continua

Patients with secondary, or symptomatic hemicrania continua (HC) may also respond to indomethacin. The HC phenotype can be observed in association with different brain diseases, in both cervical and intracranial blood vessels abnormalities, and even in lung cancer [21]. In selected patients evaluation for cervicogenic headache may be warranted.

### 4. Other primary headache disorders

A case can be made for MRI brain in all headache disorders of this section, although some with less vigor, such as typical Primary Stabbing Headache.

#### 4.1 Primary cough headache

Cranio-cervical and brain MRI, to exclude structural abnormalities, such as a Chiari 1 malformation, subtentorial tumors [1] or intermittend hydrocephalus, is recommended when headache attacks persist. This type of headache is rarely secondary to cervical or intracranial vascular pathology. MRA of brain may be helpful but MRA of the cervical arteries is not generally recommended [22]. In cough headache with tonsilar descent a dynamic MRI CSF study and/or MRI gandolinium can be necessary. MRI CSF flow measurement could be useful when low or high intracranial pressure is suspected.

#### 4.2 Primary exercise headache

In addition to brain MRI, lumbar puncture, brain MRA and MRA of carotid/vertebral arteries are also recommended to rule out subarachnoid hemorrhage and arterial dissection on first occurrence of exercise headache, assuming time to peak severity of headache is short, i.e. in minutes. Notably, the sensitivity of brain CT for subarachnoid hemorrhage approaches almost 100 % within the first hours, while the sensitivity of lumbar puncture with detection of bilirubin and MRI with hemosiderin sensitive sequences increases with time [23]. With recurrent exercise headaches these examinations should be made when warning signs occur as described in the introduction. Cardiological evaluation is recommended to exclude coronary heart disease resulting in cardiac cephalalgia. Pheochromocytoma, carcinoid, intracranial hypertension, spontaneous intracranial hypotension and cerebral venous thrombosis should be ruled out with appropriate additional investigation upon the treating physician’s decision [24]. Reversible vasospasm and/or arterial hypertension can be a diagnostic challenge as well.

#### 4.3 Primary headache associated with sexual activity

To exclude subarachnoid hemorrhage and arterial dissection in addition to brain MRI, brain MRA-venography and ultrasound/or MRA of carotid and vertebral arteries are recommended to rule out RCVs. In younger subjects, additional test of D-dimer may be useful. Upon physician’s decision a lumbar puncture and CSF bilirubin test is also considered, depending on the time of evaluation, after the episode of headache [23]. Blood tests including an erythrocyte sedimentation rate (ESR) and a CRP may be useful as well. Appropriate test to rule out reversible vasospasm and/or arterial hypertension may be needed.

#### 4.4 Primary thunderclap headache

The same exams as in section 4.3

#### 4.5 Cold-stimulus headache

No investigation is needed.

#### 4.6 External-pressure headache

No investigation is needed.

#### 4.7 Primary stabbing headache

Some experts would advocate exams as in section 4.3 in this cohort, while others would contend this disorder, in the presence of a normal clinical examination, is so typical, investigation may not be necessary.

#### 4.8 Nummular headache

Apart from brain MRI, ESR, ANF and RF may be useful [25].

#### 4.9 Hypnic headache

MRI of the brain, blood testing (including ESR, WBC and CRP), polysomnography and 24-hour blood pressure monitoring are recommended in all patients [26, 27].

#### 4.10 New daily persistent headache

A gadolinium-enhanced brain MRI with MR venography and a lumbar puncture with CSF manometry can be indicated in selected patients. Viral titers for Epstein Barr virus and cytomegalovirus may be helpful, upon treating physicians’ appraisal [28].

### 3. Notes

3.1 Gadolinium – enhanced MRI in some cases is recommended to exclude blood-brain barrier damage and leptomeningeal enhancement (e.g. low CSF pressure headache).

3.2 In the presence of symptoms suggestive of intracranial hypertension or to follow-up intracranial hypertension optical coherence tomography (OCT) of the retina may be useful [29, 30].

3.3 A person is considered over weighted when the body mass index (BMI) is higher than 25.0 and obese when BMI > 30 [http://www.cdc.gov/obesity/adult/defining.html].

3.4 Arterial dissection does not only mimic migraine but also TACs in particular [31], thus caution to exclude this condition is suggested.

3.5 Indomethacin-responsiveness has been described in secondary, or symptomatic paroxysmal hemicrania, thus pseudo-positive indomethacin test should be considered [32].

### 4. Comments

4.1 European Federation of Neurological Societies published guidelines for neuroimaging procedures in 2011. It is stated, “(i) Interictal EEG is not routinely indicated in the diagnostic evaluation of patients with headache. Interictal EEG is, however, indicated if the clinical history suggests a possible diagnosis of epilepsy (differential diagnosis). Ictal EEG could be useful in certain patients suffering from hemiplegic or basilar migraine. (ii) Recording evoked potentials is not recommended for the diagnosis of headache disorders. (iii) There is no evidence warranting recommendation of reflex responses or autonomic tests for the routine clinical examination of patients with headache. (iv) Manual palpation of pericranial muscles, with standardized palpation pressure, can be recommended for subdividing patient groups but not for diagnosis. Pain threshold measurements and EMG are not recommended as clinical diagnostic tests. (v) In adult and pediatric patients with migraine, with no recent change in attack pattern, no history of seizures, and no other focal neurological symptoms or signs, the routine use of neuroimaging is not warranted. In patients with trigeminal autonomic cephalalgia, neuroimaging should be carefully considered and may necessitate additional scanning of intracranial/cervical vasculature and/or the sellar/orbital/(para) nasal region. In patients with atypical headache patterns, a history of seizures and/or focal neurological symptoms or signs, MRI may be indicated. (vi) If attacks can be fully accounted for by the standard headache classification (IHS), a PET or SPECT scan will normally be of no further diagnostic value. Nuclear medical examinations of the cerebral circulation and metabolism can be carried out in subgroups of patients with headache for the diagnosis and evaluation of complications, when patients experience unusually severe attacks or when the quality or severity of attacks has changed. (vii) Transcranial Doppler examination is not helpful in headache diagnosis” [33]. These recommendations are in line with those presented here. Nuclear medical examinations this committee believes offer little benefit however.

4.2 In an emergency setting only CT scans are available commonly in several European countries. In this case, CT scan and CT angiography may replace MRI and MRA. This applies in particular for the cases of complications of migraine, primary exercise headache, primary thunderclap headache, or headache associated with sexual activity.

4.3 History of drug intake is highly recommended, to exclude or diagnose coexisting medication overuse headache (MOH) in all patients with chronic headache syndromes.

4.5 In general, if a headache is probable or atypical according to ICHD-3-b the threshold for additional investigations should be lower.

4.6 Psychiatric consultation and potential neuropsychological testing is recommended by some experts for almost all cases of any chronic headache disorder. Others would argue that only those patients with evident co-morbid psychiatric disorders need referral.

4.7 In patients with anxiety disorder and fear of a brain tumor, CT might be indicated for psychological reasons.

4.8 Detailed neurological examination may reveal soft neurological sings in primary headache disorders but the relevance of these sings should to be further investigated [34].
